# Perspectives and Needs Regarding Remote Monitoring Technologies Among South Asian Individuals Living With Long-Term Conditions in the United Kingdom: Semistructured Interview and Focus Group Study

**DOI:** 10.2196/82333

**Published:** 2026-05-26

**Authors:** Syed Mustafa Ali, Yumna Masood, Karen Staniland, William G Dixon, Sabine N van der Veer, Caroline Sanders

**Affiliations:** 1 Division of Informatics, Imaging and Data Science, Manchester Academic Health Science Centre, The University of Manchester, Portsmouth St, Manchester, M13 9GB, United Kingdom, +44 161 306 7876; 2 National Institute for Health and Care Research (NIHR) Applied Research Collaboration – Greater Manchester (ARC-GM), Manchester, United Kingdom; 3 Northern Care Alliance NHS Foundation Trust, Salford, United Kingdom; 4 Centre for Primary Care and Health Services Research, Division of Population Health, Health Services Research and Primary Care, The University of Manchester, Manchester, United Kingdom

**Keywords:** patient-generated health data, remote monitoring, digital inclusion, South Asian individuals, ethnic health inequalities, language barriers

## Abstract

**Background:**

South Asian individuals face a higher burden of long-term conditions while also experiencing more inequities in health care access and outcomes. Despite the potential of remote monitoring technologies to improve management of long-term conditions, South Asian individuals are less likely to engage with digital health interventions and are underrepresented in health research, partly due to language barriers.

**Objective:**

This study explored the perspectives and needs regarding remote monitoring technologies of South Asian individuals living with a long-term condition in the United Kingdom who did not have English as their first language. We used Pakistanis as an example subgroup of South Asian individuals and rheumatoid arthritis and early inflammatory arthritis as example long-term conditions.

**Methods:**

We conducted semistructured interviews and a focus group discussion with Pakistani adults diagnosed with rheumatoid or early inflammatory arthritis who did not have English as their first language. Audio-recordings were transcribed verbatim, deidentified, and analyzed thematically.

**Results:**

Seventeen adults participated in this study (n=9, 53% in an individual interview and n=8, 47% in the focus group); none of them had previous experience of remote monitoring technologies. We identified three themes: (1) the perceived value and challenges of using remote monitoring technologies for disease self-management, (2) differences in perceived needs and capacity for using remote monitoring technologies between first- and later-generation immigrants related to social determinants, and (3) the role of community and family support in using remote monitoring technologies. Participants perceived remote monitoring technologies as useful, particularly where they were dissatisfied with current health care services. Language and the role of family and community members in supporting technology use were considered important factors, but needs in these areas varied between first-generation (migrated to the United Kingdom) and second- or third-generation immigrants (born in the United Kingdom to parents or grandparents who migrated to the United Kingdom). For first-generation immigrants, these factors intersected with other social and digital determinants, such as gender and literacy, resulting in additional requirements.

**Conclusions:**

Addressing language and literacy barriers, alongside leveraging family and community support, will contribute to equitable remote monitoring technologies to facilitate self-managing long-term conditions among South Asian ethnic minority groups. Future efforts should focus on developing tailored, culturally responsive approaches, particularly for first-generation immigrants, to ensure remote monitoring technologies decrease rather than exacerbate existing ethnic health inequities.

## Introduction

South Asian individuals represent a substantial ethnic minority in many high-income countries, including the United Kingdom [1] and the United States [2]. In these settings, South Asian individuals experience a higher prevalence of long-term conditions [3-5] and related mortality [6,7] compared with their White counterparts. They are also more likely to live with multiple long-term conditions [8], but their needs often remain unmet [9,10]. These differences may partly be attributed to culturally influenced health and treatment beliefs and to language barriers that hinder understanding and engagement with treatment [11-14]. Delayed or limited access to health services further compounds poor experiences and health outcomes [13], as well as quality of life and work productivity [15].

Culture and language shape people’s perceptions of and communication about health [16,17], as well as their interactions with health care services and subsequent outcomes [18]. For South Asian individuals, language proficiency is crucial to accessing health information and services. Those with limited formal education may need interpretation support [19], which is not always available due to a shortage of interpreters and logistic complexities [20]. Specific subgroups among South Asian individuals, such as older adults and immigrant women, may face additional challenges, including misalignment between their culturally influenced health beliefs and behaviors and how health services are offered [21]. Compounding these issues is the lack of cultural confidence and competence among health care professionals [22,23], which in turn can result in poor patient and health care provider communication and a lack of consideration of sociocultural factors in care planning. For instance, managing chronic pain effectively requires sensitivity to a patient’s cultural context, yet such considerations are often overlooked, leading to suboptimal treatment decisions [24,25].

Remote monitoring technologies, such as symptom-tracking apps or connected home blood pressure monitors, have the potential to improve patient and health care provider communication when managing long-term conditions [26-28], for example, by providing visual summaries of changes in symptoms [29]. The UK National Health Service (NHS) Race and Health Observatory further recognized the potential of digital health technologies to address inequities in health service access and outcomes for ethnic minority populations [30]. However, the successful implementation of such technologies relies on patients’ digital literacy skills [31] and meaningful, digitally enabled engagement between patients and health care providers [32]. These requirements may present particular challenges for South Asian communities, where language barriers and culturally inappropriate health practices may hinder uptake and sustained use. Applying digital equity frameworks [33,34] and engaging with South Asian communities during the early stages of technology development may help address these issues and contribute to digital health solutions that are equitable, inclusive, and culturally appropriate.

However, despite this growing attention to digital health technologies, South Asian individuals have lower use rates of digital health tools within the NHS [35] and remain underrepresented in related research [36,37]. This is likely due to intersecting factors at individual and system levels, resulting in language barriers, health beliefs, behaviors [38], and challenges in accessing digital health services and research [39]. These factors leave South Asian individuals’ perspectives and needs regarding remote monitoring technologies largely unknown.

Therefore, this study aimed to explore the perspectives and needs regarding remote monitoring technologies among South Asian individuals in the United Kingdom living with a long-term condition, specifically those who do not speak English as their first language. We used Pakistanis as the largest example subgroup of South Asian individuals living in the United Kingdom and rheumatoid arthritis and early inflammatory arthritis as example long-term conditions.

## Methods

We used a narrative study design as we wanted to focus on people’s narrative accounts of their unique experiences and perceptions regarding various aspects of human interactions and culture [40]. We used the Consolidated Criteria for Reporting Qualitative Research (COREQ) checklist to report this study [41] (Checklist 1).

### Ethical Considerations

The study received ethics approval from the UK Health Research Authority South Central - Berkshire B Research Ethics Committee (22/SC/0103). All participants provided written informed consent before taking part, ensuring they understood the purpose of the study, data confidentiality measures, and their right to withdraw from the study without impact on their care and treatment. All participants received compensation for their time. The collected data have been anonymized to safeguard participants’ information. The study was conducted in accordance with the Declaration of Helsinki and its later amendments.

### Study Context

We used rheumatoid arthritis as the example long-term condition for our study because it is more prevalent among South Asian individuals [42]. They also face significant delays in being diagnosed with rheumatoid arthritis [43] and are less likely to receive treatment [9]. Similar to conditions such as asthma, heart failure, and inflammatory bowel disease, management of rheumatoid arthritis relies on patient reports of symptoms and flares. Many patients struggle to describe these accurately during their brief and infrequent outpatient consultations [44], which may lead to incomplete and inaccurate information, poor treatment decisions, and ultimately worse outcomes [45,46]. This may be harder for people who do not have English as their first language. Remote monitoring technologies may help to address this by enabling all patients to track their symptoms at home using a smartphone app and share this symptom data with their health care team for discussion during consultations. As an example technology for this study, we used the Remote Monitoring of Rheumatoid Arthritis (REMORA) symptom-tracking smartphone app, which served as a prompt during interviews and focus groups. The app is currently being evaluated as part of a complex intervention in a randomized controlled trial [47].

### Study Participants and Recruitment

We recruited participants using a purposive sampling method. People were eligible to take part if they were aged 18 years or above, did not have English as their first language, self-identified as Pakistani, had a diagnosis of rheumatoid arthritis or early inflammatory arthritis, and were able to give informed consent. We focused on Pakistanis as a South Asian subgroup because they form the largest ethnic minority in the United Kingdom [1], and we had 2 research team members from the same background: SMA, a male digital and public health researcher, and YM, a female qualitative health service researcher. Both had Urdu as their first language and had experience working with the Pakistani community in the United Kingdom. SMA and YM were mindful of their possible influence on the data collection, analysis, and interpretation, and they used reflexivity to bring insights, which were relevant for addressing the study objectives.

We identified and recruited potential participants via a rheumatology outpatient clinic and a community center for South Asian women in Greater Manchester. Local research nurses and a community center manager approached people to introduce the study, share or read the participant information sheet, and obtain consent to contact from those who were interested. One researcher (YM) contacted potential participants to determine whether they had any questions and were willing to participate, after which they were asked to provide written informed consent. Recruitment continued until data saturation was achieved, with no new ideas emerging from the interviews.

### Data Collection and Analysis

**Figure 1.**
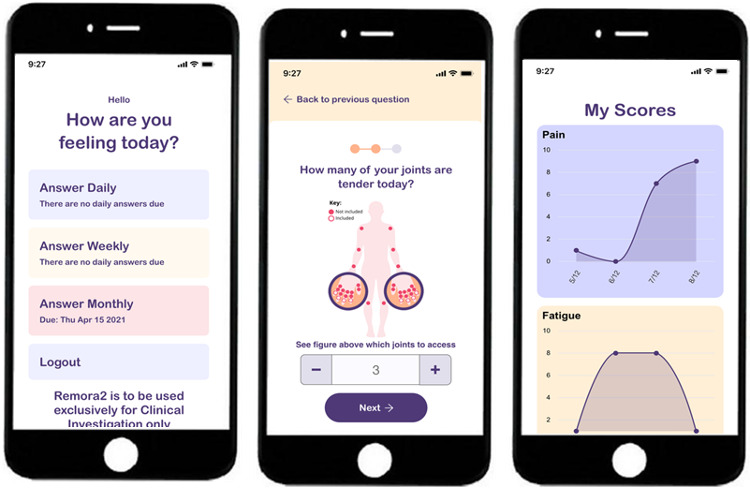
Screenshots of the Remote Monitoring of Rheumatoid Arthritis (REMORA) app, an example remote monitoring technology (Copyright University of Manchester).

We used a brief questionnaire to collect data on participants’ age, gender, language proficiency, and their diagnosis. People were invited to participate in either a focus group or an individual interview, whichever they preferred. We conducted interviews and a focus group discussion with the help of topic guides (MultimediaAppendices 1  2), which were developed based on published literature [34,37,39,48] and input from patient representatives (KS and others). Topics included experiences of living with rheumatoid arthritis or early inflammatory arthritis, experiences of health care services, experiences of using digital technologies, views on symptom monitoring, and views on using digital health care technologies, including potential benefits or barriers. Previous studies have highlighted the importance of the wider context in which people manage their health care problems, including any social support and existing health care arrangements, when researching perspectives regarding implementation of new health care technologies [42]. Consequently, interviews and the focus group began with a discussion of this wider context before focusing on views about remote monitoring technologies. The topic guides provided a structure for the interviews and focus group discussion while leaving flexibility for participants to introduce topics that researchers had not considered. In addition to the topic guides, we used screenshots from the REMORA app as visual prompts to give participants an idea about what a remote monitoring technology could look like (Figure 1).

Individual interviews were conducted in Urdu or in English, whichever language participants preferred. The focus group discussion was in English, as participants were bilingual and were comfortable sharing their thoughts in English. All data collection happened in a community setting familiar to participants, which facilitated a more comfortable and culturally sensitive interaction. All interviews and the focus group were audio-recorded and transcribed verbatim in the language in which they were conducted. The interviews lasted approximately 45 minutes, while the focus group discussion lasted approximately 90 minutes.

SMA and YM independently reviewed transcripts line by line and assigned codes using NVivo software (version 12; Lumivero) to manage the data. For interviews in Urdu, SMA and YM coded the Urdu transcripts and selected and translated relevant quotes into English through discussion. Selected and translated quotes were then discussed with the wider research team (CS, KS, SMA, SNvdV, and YM) to guide iterative coding, thematic analysis, quote selection, and interpretation. We also shared a summary of the findings with our patient representatives to sense-check our interpretations. Finally, SMA and YM double-checked translations of selected quotes used to illustrate themes in the manuscript.

## Results

### Overview of Participants

Table 1 shows the characteristics of the 17 participants, of whom 10 (59%) participated in an individual interview (9 in Urdu and 1 in English). Most were women (n=16, 94%), aged 60 years and above (n=11, 65%), migrated to the United Kingdom during their life (hereafter referred to as first-generation immigrants; n=15, 88%), bilingual (proficient in speaking and understanding English but with limited proficiency in written English language; n=16, 94%), and living with multiple long-term conditions (hypertension and diabetes were most common; n=11, 65%).

**Table 1.** Characteristics of participants (n=17).

Variables	Value, n (%)
Sex	
Female	16 (94)
Male	1 (6)
Age group (y)	
45‐59	6 (35)
≥60	11 (65)
First-generation immigrant^a^	
Yes	15 (88)
No	2 (12)
Working	
Yes	7 (41)
No	10 (59)
Recruitment route	
Community center	14 (82)
Outpatient clinic	3 (18)

a Defined as having migrated to the United Kingdom during their life.

Perspectives and needs related to remote monitoring technologies were explored within a wider context of managing rheumatoid arthritis or early inflammatory arthritis and experiences of health care services. None of the participants had prior experience using any remote monitoring technology. We identified three main themes that reflected participants’ views regarding remote monitoring technologies and were developed through an iterative process of analysis: (1) perceived value and challenges of using remote monitoring technologies for disease self-management, (2) differences in perceived needs and capacity for using remote monitoring technologies between first- and later-generation immigrants related to social determinants, and (3) the role of community and family support for using remote monitoring technologies. We described the themes in more detail in the following sections, illustrated with participants’ quotes and characteristics (individual interviews only).

### Perceived Value and Challenges of Using Remote Monitoring Technologies for Disease Self-Management

Participants perceived both challenges and value in using remote monitoring technologies for disease self-management. Particularly, their self-management practices or lay remedies and their experiences with health care services provided a relevant context for discussing the potential value of integrating remote monitoring technologies in their care.

The use of culturally influenced lay remedies was reflected strongly in people’s health beliefs and behaviors regarding managing their condition. All participants mentioned the use of one or more lay remedies, ranging from consuming turmeric, garlic, vinegar, honey, ginger, and herbs to applying massaging oil and turmeric paste on painful sites. Participants also mentioned incorporating lifestyle modifications, such as exercise, yoga, and changes in diet (eg, consuming more vegetables) to manage their condition.

*I use herbal products too...sometimes I ask someone to bring them from Pakistan or sometimes I get them from Birmingham. Consuming them makes me feel better.*

[Female, aged 70‐80 years, retired, first-generation immigrant]

*My condition gets worse when I have flare ups...for that time...someone told me to apply black seed oil on my heels...I also add vicks to it...it feels better.*

[Male, aged 50‐60 years, working, first-generation immigrant]

Many participants reported unsatisfactory experiences with health care services, seemingly stemming from a long and difficult diagnostic process, a lack of treatment options being offered, delayed appointments with health care professionals, and limited consultation time. One of the participants said:

*Firstly, you don’t get an appointment...if you get it...then they are not interested in knowing about what has been happening with you.*

[Female, aged 60‐70 years, retired, first-generation immigrant]

They also shared their expectations about support between clinical consultations:

*I think so between consultations there should be a person who can advise how to manage your pain and your arthritis. This can help a lot, because not everyone is alike*.

[Female, aged 70-80 years, retired, first-generation immigrant]

Participants also mentioned how memory affected their day-to-day living and how this might have affected the quality of their consultations with clinicians. One participant described it as follows:

*I go to my appointment myself...I leave home, and when I reach for my appointment, I forget what I am here for.*

[Female, aged 50-60 years, working, first-generation immigrant]

Following these previous experiences, participants acknowledged the potential value of remote monitoring technologies in aiding patient and health care provider interaction, helping patients between consultations, scheduling appointments, and helping them remember and communicate important information about flares, their underlying causes, and their frequency during clinical consultations. One of the participants described how the use of remote monitoring technology could help in memorizing key information about their conditions:

*[E]ven if I open that app and start using it, I may forget what I wanted to do...but then it would also refresh my mind. If you have already put some information in it then it won’t go anywhere*.

[Focus group participant]

Remote monitoring technologies could potentially benefit patients by capturing changes in their pain and guiding them in managing their conditions better, as described by one of the participants:

*It [remote patient monitoring] can help you monitor your condition, if it is getting better or worse, and what are the underlying causes...and what are the alternatives...if your condition is getting better, then how can you improve it further and if it is getting worse then you can identify if a consultation or help is needed.*

[Female, aged 60-70 years, retired, first-generation immigrant]

Recognizing the potential benefits of remote monitoring technologies, all participants expressed positive views about sharing their data with health care professionals. However, one of the participants raised concerns about data sharing:

*Well...it depends where it [data] is going, where it’s going to be used, or how far will it go...I don’t know.*

[Female, aged 70-80 years, retired, first-generation immigrant]

In addition, some older women expressed a lack of motivation for learning to use new digital tools. One woman aged >70 years, who opposed using remote monitoring technologies, shared her frustration regarding trying different tools or approaches for managing rheumatoid arthritis:

*I have accepted now that this the way I am going to live my life...initially I was advised to maintain a diary, which I used to do. Now I have threw everything away...that I will not do anything now...I feel like I am fed up of all this.*

[Female, aged 70-80 years, retired, first-generation immigrant]

When asked how barriers to accessing and using remote monitoring technologies among Pakistani people could be addressed, participants suggested some technology features such as in-app reminders to submit symptom scores, questions or instructions presented in translated and/or audiovisual formats, information explaining how to interpret symptom summary reports, and biometrics-enabled login instead of using passwords. Participants shared challenges or difficulties related to their experience of using technologies:

*The password is the worst...when you have to make it and they say it’s not right, do this, there’s no capital. I hate passwords. I have to get my sons to do it.*

[Focus group discussion]

### Differences in Perceived Needs and Capacity for Using Remote Monitoring Technologies Between First- and Later-Generation Immigrants Related to Social Determinants

Participants suggested differences in needs and capacity for using remote monitoring technologies between first- and second- or third-generation (ie, those born in the United Kingdom to parents or grandparents who migrated to the United Kingdom during their lifetime) Pakistani immigrants and related this generational aspect to other social and digital determinants of health, such as gender. For example, one of the participants referred to gender norms and said:

*Women are usually at home and are not exposed to technology, and in our culture, it’s very common that women are least engaged with new technologies.*

[Male, aged 50-60 years, working, first-generation immigrant]

Limited proficiency in English was mentioned by participants as a key potential barrier to using remote monitoring technologies, mainly for first-generation immigrants. Among these immigrants, participants suggested young immigrant women (ie, who moved to the United Kingdom at a young age) were a subgroup at greater risk of developing inequities because of their language barriers. For example, some young immigrant women can only communicate effectively in their local language (such as Punjabi or Pothwari) rather than in Urdu, the national language of Pakistan. One of the participants described it as:

*The ladies group that I attend regularly [organized by local charities]...most of the group members don’t know how to speak English...they sometimes find it hard to communicate in Urdu...these are the ladies who came to the UK for marriage when they were very young, and they typically lack good education and digital skills.*

[Female, aged 60-70 years, retired, first-generation immigrant]

One of the participants suggested how language requirements could be addressed:

*[Referring to the REMORA app] see the content of the app in English, which is a barrier. This should be translated into Urdu...may be in audio format. Certain words such as tiredness and others in the app can be best described through images or visuals.*

[Male, aged 50-60 years, working, first-generation immigrant]

There were also different language requirements between first- and second-generation immigrants, suggesting that not every individual with a Pakistani background would require Urdu to access digital health tools and resources (ie, understanding written materials or content). For example, second-generation immigrants can understand spoken Urdu but may not be able to read Urdu. Similarly, first-generation immigrants are proficient in spoken English but may struggle to read English. One of the participants described it as follows:

*Having the app in English and Urdu languages will be helpful...our kids understand Urdu...but if you speak to them in Urdu, they feel shy in answering in Urdu, but they do understand it...they just not speak Urdu.*

[Female, aged 60-70 years, retired, first-generation immigrant]

Participants considered second- or third-generation immigrants to be more educated and digitally skilled:

*This second generation is very educated and intelligent. They are well settled and capable to do anything. They are capable to use digital health technologies too.*

[Male, aged 50-60 years, working, first-generation immigrant]

Among second- or third-generation immigrants, participants also perceived an emerging need for digital health tools because Pakistani people are being diagnosed with rheumatoid arthritis or early inflammatory arthritis at younger ages. Acknowledging the hesitation among older adults, one of the participants highlighted the need for older adults or first-generation immigrants to use digital health tools for managing their health conditions:

*...if you can’t look after yourself who’s going to look after the rest of the family? So, take the time out for yourself. I encourage other ladies to have a free computer lesson. It’s a big help to them. But they are reluctant to go, they are scared to go.*

[Focus group participant]

According to participants, most first- and second- or third-generation immigrants either lived together in the same household or remained in close contact to support each other in health and non–health-related matters. Related to this, participants identified older women living alone as an example of a subgroup at risk of inequities because of their living and social circumstances. One of the older women said:

*There are women in their older age, who live alone, their husbands are dead, their kids are settled elsewhere, so normally they are left with their own challenging circumstances...I personally think that women of my [older] age are either not interesting in using digital health technologies or they don’t have enough resources to support the use of these technologies.*

[Female, aged 60-70 years, retired, first-generation immigrant]

### Role of Community and Family Support for Using Remote Monitoring Technologies

Participants acknowledged family and communal values of helping each other, particularly older people, individuals living in challenging circumstances, and those with less education. For example, one of the participants said about older adults:

*Older people normally would not be able to use these tools [remote monitoring technologies] on their smartphones. Unless there are community groups...just like the one I attend...where someone sits with them and explains and shows them*.

[Male, aged 50-60 years, working, first-generation immigrant]

Similarly, participants described how some women got help from local community groups, where they received support for day-to-day living and managing health issues. In addition to community support, children and grandchildren could provide family support for their parents and grandparents to handle their day-to-day affairs and manage health issues. For example, they could find and share relevant health-related information or show them how to search for relevant information online themselves (eg, YouTube videos, which participants mentioned as a common source of information, often shared by their family or friends). One of the participants said:

*I am not very educated...so kids can help us in recording important information in the app so that doctor could access that information. Otherwise, I would not be able to remember and communicate this information during consultation.*

[Female, aged 50-60 years, working, first-generation immigrant]

Sometimes, help available from children and grandchildren was a reason for older adults to lose interest in learning new skills and tools that could otherwise enable them to manage their own conditions better without receiving help from their children. One of the participants said:

*I am not interested in learning how to use the [remote patient monitoring] app, when all such needs are fulfilled at home by family members.*

[Female, aged 60-70 years, retired, first-generation immigrant]

Finally, participants proposed different activities and resources that might be helpful for patients to improve their health and digital literacy, particularly regarding the use of remote monitoring technologies. For example, disease-specific awareness sessions are regularly conducted by local physicians in community settings. Participants proposed organizing similar sessions to promote the potential value of remote monitoring technologies and train people how to use them effectively. In addition to in-person sessions, participants also proposed audiovisual content to help enable them to use such technologies. Group exercise sessions were also suggested, as one of the participants described the difficulty they faced:

*Say, a physiotherapist gave you exercise, then they say go and just do it. There is no follow-up, there is nothing. You are just left to your own devices. If there is something that people every so often kept on top of called you in, there was some groups or sessions, and it’s like how you are doing, and has it worked or not worked?*

[Female, aged 70-80 years, retired, first-generation immigrant]

## Discussion

### Principal Findings

This qualitative study explored perspectives and needs regarding the use of remote monitoring technologies for self-managing long-term conditions among South Asian individuals living in the United Kingdom who did not speak English as their first language. Study participants were dissatisfied with health care services and perceived remote monitoring technologies as a potential solution to these issues. For example, they believed these technologies could help address memory problems, enrich patient and health care provider communication, and help them feel supported between consultations. However, some subgroups (such as older women living alone in challenging circumstances or young immigrant women with language barriers and poor educational backgrounds) were considered at greater risk of developing further inequities if remote monitoring technologies are introduced in care pathways. Moreover, first-generation immigrants were expected to face more inequities than later-generation immigrants because of differences in intersecting social and digital determinants. Participants highlighted how family and communal values within the South Asian community could be leveraged to guide the development of support for people using remote monitoring technologies, facilitated by audiovisual content and in-person awareness sessions.

### Relation to Other Studies

Despite recognizing the perceived benefits of remote monitoring technologies, common authentication methods (eg, passwords), along with a lack of motivation to learn how to use these technologies, may create accessibility barriers [49], as reported by participants in our study. In addition to addressing these accessibility barriers, the provision of timely and personalized care may further promote sustained engagement with remote monitoring technologies [28].

Our study showed intergenerational differences in perspectives and needs regarding the use of remote monitoring technologies to support South Asian individuals in managing their condition. In keeping with Aldosari et al [39], our study highlighted that literacy levels and language requirements varied between first- and second-generation immigrants. This supports previous research advocating language proficiency as an important determinant for equitable access to and use of digital health technologies for South Asian individuals [39] and for ethnic minority groups more generally [50,51]. While technological capabilities to translate English content are improving, people with language barriers may still prefer face-to-face communication [38] and therefore remain reliant on available interpretation support via professional interpreters or informal carers [52]. In turn, this influences their trust in digital health research and services [37,53], making it crucial to explore and address language requirements to ensure equitable and inclusive digital health research and technologies.

We also found that the role of family and community members is important in supporting the use and adoption of remote monitoring technologies. Their role is increasingly being recognized in facilitating South Asian individuals’ access to digital health tools and services [38,54] and building their skills and trust in using digital health technologies [55,56]. Particularly in the context of multigenerational households, living arrangements may improve digital literacy and health care access [57] and help navigate usability issues related to digital health technologies [58]. This dependence might not necessarily be perceived as negative, as in Asian cultures more broadly, interdependence tends to be valued (rooted in filial piety, a concept where social norms shape intergenerational relationships [59]). However, we also found that the availability of support from family carers (eg, children or grandchildren) could have a simultaneous negative impact on people’s motivation to obtain digital skills. This aligns with findings from another study in South Asian individuals living with cardiometabolic diseases, suggesting that family members and South Asian community members acted as both positive and negative drivers for adopting healthier versions of cultural food [38]. Overall, this suggests that social context could be leveraged to enhance the adoption of remote monitoring technologies, but future research should further explore how family and community support moderate interdependent relationships and ultimately people’s engagement with digitally enabled health care services.

### Limitations

One of our eligibility criteria was that participants did not have English as their first language. Our aim was to recruit a sample at risk of experiencing language barriers when engaging with digital health technologies and research. Overall, in the United Kingdom, around 20% of people who do not have English as their main language report limited or no English language proficiency [60]. However, most participants in our study were able to speak English very well and did not consider language an important barrier for themselves. Although they highlighted the English language as a potential barrier for some other first-generation immigrant women, our sample did not allow in-depth exploration of perspectives from those with more limited English language proficiency.

Most study participants were recruited via a community-based organization that organized regular health-related activities (eg, awareness sessions and exercise sessions). This may partly explain why community support was identified as a potential enabler for using remote monitoring technologies. Patients recruited through other routes or less health-focused community-based organizations might have had different perspectives on the role of community-based resources in addressing ethnic health inequities.

### Implications for Practice and Research

Researchers and technology developers should consider proficiency in reading and writing English or people’s native language as an important determinant of health when designing equitable remote monitoring technologies for end users from South Asian ethnic minority groups. This should include exploring the language proficiency of end users’ family members (eg, children or grandchildren) to examine if and how information should be offered to ensure it is accessible to multiple generations, enabling both the end user and their “supporters” to engage with digital health tools and resources. Requirements to involve family carers are also reflected in NHS England guidance on access to health services, including the implementation guidance on accessible information standards [61] and the framework for action on digital inclusion [62]. However, the literature provides limited specific examples of how language needs are addressed in practice. Therefore, future research should examine how aspects of language requirements for digital health technologies can be effectively implemented, particularly within the context of multigenerational households. These explorations should form part of understanding people’s wider social context and of proactively involving family carers in co-designing remote monitoring technologies, as has been done previously in the context of dementia [54] and diabetes [63]. While digital skills-building training, as well as educational and cognitive-behavioral programs, have been developed for carers to enhance their pain-related knowledge and self-efficacy, robust evidence of their effectiveness remains limited. Therefore, future research should focus on strengthening this evidence base [64,65]. Overall, involvement of carers will support the development of more equitable remote monitoring technologies that contribute to better health outcomes among South Asian ethnic minority groups.

### Conclusions

Remote monitoring technologies have the potential to enhance care for South Asian individuals by addressing language barriers, cultural needs, and issues of trust. Language barriers often intersect with other social and digital determinants, such as age, educational attainment, and digital health literacy. This creates further obstacles, especially for first-generation South Asian immigrants, who may require additional support. In this context, the role of family carers and the wider community in supporting access to and use of remote monitoring technologies is particularly important. Researchers and technology developers must therefore consider the social and cultural environments in which people from South Asian minority backgrounds live to ensure that remote monitoring technologies are inclusive, accessible, and capable of addressing existing health inequities for those living with long-term conditions.

## Supplementary material

10.2196/82333 Multimedia Appendix 1 Topic guide for interviews.

10.2196/82333 Multimedia Appendix 2 Topic guide for workshop.

10.2196/82333 Checklist 1 COREQ checklist.

## Abbreviations

COREQ: Consolidated Criteria for Reporting Qualitative Research
NHS: UK National Health Service
REMORA: Remote Monitoring of Rheumatoid Arthritis
