# Supplementary material for: Perspectives and Needs Regarding Remote Monitoring Technologies Among South Asian Individuals Living With Long-Term Conditions in the United Kingdom: Semistructured Interview and Focus Group Study
Source: JMIR Hum Factors. 2026 May 26;13:e82333. doi: 10.2196/82333 (PMC13211867; doi:10.2196/82333)
Supplement: Multimedia Appendix 1 [file humanfactors-v13-e82333-s001.pdf]

<<Trust and study logo>>

Introduction

- Thank you for agreeing to take part
- Explanation of research & aims
- Details of participation
- Voluntary (can withdraw any time) and confidential
- Audio recording and data protection
- You do not have to discuss anything that will make you feel uncomfortable
- Any questions?
- Start recording.

History of rheumatoid arthritis and self-management

- Can you tell me about your 'rheumatoid arthritis, when you first experienced problems with him/her and what happened?
- What do you do to manage your rheumatoid arthritis symptoms?
- What resources do you use other than health services for managing your arthritis?
- What is most important to you in terms of support or help with your arthritis?

Previous and current use of health services and interactions with professionals

- What kinds of things are most important to you when you consult health professionals about management of arthritis (communication, confidence in knowledge, information, advice etc)?

Monitoring of Symptoms

- Would you say you actively monitor and/ or record changes in your symptoms?
- What symptoms do you think have the most impact on how well or unwell they feel?

Views and experiences of technologies to assist in managing condition

- Do you currently use a smartphone, tablet or computer?
- How often do you use these and for what type of things (e.g. email, shopping? social networking)
- If not, do you have any concerns about using a smartphone?

- Have you ever used the internet to find information or support in relation to your rheumatoid arthritis? – has this been helpful?
- Have you any experience of using other health related apps? If so, can you give examples and describe how you found using them?

Views and opinions on using a mobile phone to collect health data and the potential value

- What are your views about storing and sharing health data collected by monitoring symptoms etc by mobile phone?
- What are your views about collecting information about peoples' position and movements to monitor physical activity and functioning?
- Do you have any concerns about collecting health data by mobile phone?
- What do you think are the benefits of this type of monitoring?
- If you were to collect health data for clinical purposes, would you be willing to share an anonymised copy of the data for research?
- Would you be willing for this data to be linked to your anonymised electronic medical record for research purposes?

Demonstration of initial ideas for the app

- Slides to show initial questions and scoring system
  - ➢ What are your views about the questions (wording and scoring)?
  - ➢ Are any questions problematic?
  - ➢ Are there other questions that should be asked?
- Show images of potential interface designs (from our previous apps – uMotif/ ClinTouch) and allow respondent to try using demonstrator versions on mobile phones
- ➢ What aspects of the app design do you like or not like and why?
- Demonstrate additional components and features e.g. home screen, information, medication reminders, daily diary
  - ➢ What features do you like or not like and why?
  - ➢ Do you think you would use all of these?
  - Demonstrate versions of feedback reports to summarise their scores
- ➢ Which graphs/ summaries are most useful and why?
  - General use
- ➢ How do you think you will find using this?
- ➢ Do you think you will you be happy to complete the app every day (how many times)?
- ➢ Do you anticipate any problems using the app?
- ➢ Do you have any other comments or questions about using this, or about the app?
- ➢ How can we make this app culturally appropriate for the Urdu speaking patients?

Thank you
