# Supplementary material for: Perspectives and Needs Regarding Remote Monitoring Technologies Among South Asian Individuals Living With Long-Term Conditions in the United Kingdom: Semistructured Interview and Focus Group Study
Source: JMIR Hum Factors. 2026 May 26;13:e82333. doi: 10.2196/82333 (PMC13211867; doi:10.2196/82333)
Supplement: Multimedia Appendix 2 [file humanfactors-v13-e82333-s002.pdf]

<<Trust and study logo>>

REMORA2 Interview Schedule for focus group

1. Re-introduce self and purpose of focus group

2. Check with participant:

- That they are still willing to be interviewed, and to be audio recorded
- Remind them it will take approximately 60 to 90 minutes

3. Remind participant that:
1. Their responses will be kept confidential, and quotes used in the results will not identify them as an individual.
2. They can change their mind about taking part in the study and stop the interview at any point.

4. Ask if the participant has any questions.

5. Start recording.

Section 1: Background history of RA/RMD

1. Could you describe your experience of undergoing RA/RMD treatment?

Prompt: Have you found the current treatment have helped to improve your condition? Issues /benefits? Expectations vs. lived experience.

2. Can you tell me about your current management of RA/RMD?
3. How do you feel about using digital technology like an app for monitoring your condition?
4. From you experience or viewpoint what aspects of the app or digital technology can facilitate or cause barriers to managing your condition?

Section 2: Workshop and researcher prompts-

Overview of REMORA -app demonstration and existing supporting materials. Ask about:

- Views/ experiences of remote symptom monitoring
- Views about existing materials to support engagement with REMORA
- Any gaps, problems with materials

Overview of barriers - findings from the qualitative research and ideas for methods and materials for supporting inclusion (to be co-designed with our public involvement group, and tailored to be relevant to workshop participants). Invite discussion on:

- Research findings on barriers to digital engagement
- Methods for supporting inclusion developed in partnership with public contributors
- Any additional ideas for supporting digital inclusion

Summary of methods discussed and consensus exercise to decide on methods and materials for supporting inclusion to be developed and tested

Debrief

1. Tell participant that the digital recorder is now being switched off.
2. Thank participant for taking part in the interview.
3. Revisit consent
4. Ask if the participant has any questions about the study.
5. Let them know that you will be sending all participants a summary of study findings.
6. Thank participant again for taking part in the interview.
